# Single‐Atom Alloys for the Electrochemical Oxygen Reduction Reaction

**DOI:** 10.1002/cphc.202000869

**Published:** 2021-02-03

**Authors:** Matthew T. Darby, Michail Stamatakis

**Affiliations:** ^1^ Thomas Young Centre and Department of Chemical Engineering University College London, Roberts Building Torrington Place London WC1E 7JE UK

**Keywords:** Single-atom alloy, electrocatalysis, fuel cells, density functional theory, ORR

## Abstract

Single‐atom alloys (SAAs) consisting of isolated transition‐metal atoms doped in the surface of coinage metal hosts exhibit unique catalytic properties, harnessing the high activity of the dopant metals with the selectivity of the coinage metal hosts. Here we use density functional theory (DFT) to study SAAs comprised of Ni, Pd, Pt, Co and Rh doped into Ag and Au hosts, as candidate electrocatalysts for the oxygen reduction reaction (ORR) in proton‐exchange membrane (PEM) fuel‐cells. Our calculations reveal that the PdAu SAA exhibits a slightly lower theoretical overpotential, enhanced selectivity for 4‐e^−^ ORR, and tolerance to CO‐poisoning compared to Pt(111). While the number of active sites of PdAu SAA is lower than that of Pt(111), the aforementioned desirable properties could bring the overall catalytic performance thereof close to that of Pt/C, indicating that the PdAu SAA could be a viable material for electrocatalytic ORR in PEM fuel‐cells.

## Graphical abstract

Single‐atom alloys (SAAs) comprise an exciting class of ultra‐dilute alloys, whereby the minority metal is atomically dispersed and exhibits unique catalytic properties. For electrocatalytic applications, the PdAu SAA is shown to exhibit a slightly lower theoretical overpotential, enhanced selectivity for 4‐e^−^ oxygen reduction reaction, and CO‐poisoning tolerance compared to Pt(111).

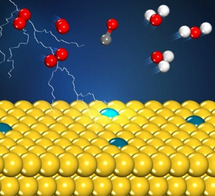

## Introduction

Key to the operation of a low temperature proton exchange membrane (PEM) fuel cell is the cathodic oxygen reduction reaction (ORR), which electrochemically reduces oxygen to water in a four electron (e^−^) process. This reduction facilitates the generation of an electric potential which is most commonly used to produce mechanical work for transportation vehicles, or as a portable and stationary emergency backup power. Despite significant effort being expended in the search for novel ORR catalysts, currently the most effective materials for catalysing the ORR are pure Pt and alloys thereof.[^1^, ^2^] However the scarcity and high cost of these metals, as well as issues with their stability and poisoning have limited the overall efficiency of PEM devices, precluding their widespread commercialisation.[^3^, ^4^] Thus, in order to reach a stage whereby PEM fuel cells are viable for practical and general use, it is essential to overcome the significant hurdle of developing novel materials that are at least as active as Pt‐based metals, but have lower cost or higher durability under ORR conditions.[^1^, ^2^, ^3^, ^4^]

One highly promising strategy that is being adopted by the catalysis community in the search for alternatives to platinum group metal (PGM) materials, is to dope an unreactive host material with trace amounts of PGMs.[^5^, ^6^, ^7^, ^8^, ^9^, ^10^, ^11^, ^12^, ^13^, ^14^] This approach minimises the amount of rare and expensive metals and yields isolated single atoms that are integrated into the surface host matrix. For several important chemistries including hydrogenation, C−H activation and C−C coupling reactions, single atom alloys (SAAs) exhibit enhanced activity and selectivity over their monometallic host and dopant metals, respectively, as well as high tolerance to catalytic poisoning.^[15]^

In our previous theoretical studies we have screened the reactivity of SAAs towards several industrially relevant chemical species including water, hydroxyl and oxygen adatoms, which are also intermediates in the ORR.[^11^, ^16^] Our calculations revealed that oxygen and hydroxyl bind weakly to Pd‐, Pt‐, Rh‐ and Ni‐doped SAAs based on Ag and Au host metals, and in some cases notably weaker than Pt(111), the model catalyst for the Pt/C electrode. Since weak binding of these species is a common descriptor for good ORR performance,^[17]^ we propose that these materials may exhibit high catalytic activity. Additionally, our calculations have shown that SAAs often bind CO much more weakly than pure PGM materials, thereby exhibiting enhanced CO tolerance.[^18^, ^19^] Though using Au and Ag to replace a large quantity of Pt in an ORR catalyst will not yield significant economic advantages and will reduce the number of active surface sites by 20‐ to 100‐fold, the combination of enhanced site activity, selectivity and resistance to poisoning could potentially lead to synergistic effects that would result in improved overall ORR behaviour of SAAs over traditional Pt‐based materials.

In light of this, we present a DFT based study that elucidates the thermodynamics of the ORR on Ag‐ and Au‐based SAAs. We have thus investigated SAAs comprised of Ni, Pd, Pt, Co and Rh, doped as single‐atoms into the (111) surfaces of FCC‐packed Ag and Au hosts. For these surfaces we consider their interaction with ORR intermediates (O_2_*, OOH*, O* and OH*) in order to evaluate the potential dependence of the ORR thermodynamics and use the limiting potential as a metric for activity. We further evaluate the selectivity of water over peroxide formation and identify the most probable pathways for ORR on each surface. Finally, we use kinetic Monte Carlo (KMC) simulations to assess the extent of CO poisoning on SAAs compared to Pt(111). Ultimately, the results presented in this manuscript highlight promising new SAA materials for catalysing the ORR, motivating their experimental development and testing towards innovative solutions for future electrochemical technologies.

## Computational Methodology

### Density Functional Theory Calculations

We have performed periodic DFT calculations using the Vienna *Ab initio* Simulation Package (VASP)[^20^, ^21^, ^22^, ^23^] version 5.4.1 under a continuum solvation model as implemented in VASP‐sol.[^24^, ^25^] The projector augmented wave (PAW) method was used to model the core ionic potentials.[^26^, ^27^] The generalised gradient approximation and specifically the functional developed by Perdew, Burke and Ernzerhof (PBE) was used to approximate the exchange‐correlation.^[28]^ We used a 3×3×5 slab unit cell whereby we fully relaxed the top‐most two layers while we fixed the bottom three layers at the PBE bulk FCC lattice constant of the corresponding metal (for SAAs, we used the host material lattice parameters of 4.17 Å and 4.18 Å for Ag and Au, respectively). For calculations on SAAs, a single surface host atom was replaced by a dopant atom giving a surface loading of 1/9. An approximate vacuum region of 15 Å above the surface was used in order to minimize periodic interactions in the z‐direction. 7×7×1 and 11×11×1 Monkhorst‐Pack k‐point meshes were used to sample the Brillouin zone for geometry optimisation and density of states calculations, respectively. The plane‐wave kinetic energy cut‐off was set to 400 eV. To aid with convergence, we employed Methfessel‐Paxton smearing with a width of k_B_T=0.1 eV; the final electronic energy is extrapolated to k_B_T=0 eV. Electronic self‐consistency was ensured up to a tolerance of 10^−7^ eV. To locate stable configurations in the potential energy surface, we performed conjugate gradient minimization of the Hellmann‐Feynman forces on all atoms to within a tolerance of 10^−2^ eV ⋅ Å^−1^. Vibrational frequencies were calculated using a harmonic approximation with finite displacements of 0.02 Å. For calculations in solvent, the dielectric constant was set to 78.4, corresponding to that of water. Transition state searches were performed using the dimer formalism of Jónsson and Henkelman.^[29]^ To validate that states found correspond to first order saddle points, we performed vibrational frequency analysis.

### Computational Hydrogen Electrode Model

We make use of the CHE model^[30]^ to study the two and four $e^{-}$ ORR pathways. The latter is the most desirable ORR pathway for a catalyst operating in a PEM fuel cell and can be summarised by the following equations (* indicates surface‐bound adspecies):

(1) $$O_{2\left(g\right)}+{}^{*}↔{O_{2}}^{{}^{*}}$$

(2) $${O_{2}}^{{}^{*}}+\left(H^{+}+e^{-}\right)↔{OOH}^{{}^{*}}\;$$

(3) $${OOH}^{{}^{*}}+\left(H^{+}+e^{-}\right)↔O^{{}^{*}}+{H_{2}O}_{\left(g\right)}$$

(4) $$O^{{}^{*}}+\left(H^{+}+e^{-}\right)↔{OH}^{{}^{*}}$$

(5) $${OH}^{{}^{*}}+\left(H^{+}+e^{-}\right)↔\;{}^{*}+{H_{2}O}_{\left(g\right)}$$

(6) $$Overall:\;O_{2\left(g\right)}+4\left(H^{+}+e^{-}\right)↔{{2H}_{2}O}_{\left(g\right)}\;$$

The associative four $e^{-}$ ORR pathway given by steps 1–5 involves the concerted molecular adsorption of O_2_ before reduction to OOH*.

An alternative pathway, which is referred to as the four $e^{-}$ dissociative pathway, involves the scission of the O_2_ dimer to form two surface O adatoms:

(7) $${O_{2}}^{{}^{*}}+\;{}^{*}\;↔{2O}^{{}^{*}}\;$$

After step 7, this pathway is completed by reduction to water through steps 4 and 5, and the overall reaction is the same as that of the associative pathway: reaction (6).

Finally, a less desirable pathway for application in PEM fuel cells exists, which involves two electrons. This two $e^{-}$ ORR pathway may compete with the complete four $e^{-}$ reduction and is summarised as:

(8) $$Overall:\;O_{2\left(g\right)}+2\left(H^{+}+e^{-}\right)↔{H_{2}O_{2}}_{\left(g\right)}\;$$

The two $e^{-}$ ORR pathway initially proceeds via steps 1 and 2, yielding the OOH* intermediate. An additional step to form hydrogen peroxide concludes the two $e^{-}$ ORR pathway:

(9) $${OOH}^{{}^{*}}+\left(H^{+}+e^{-}\right)↔{H_{2}O}_{2\left(g\right)}$$

Using the CHE model, we calculate the free energy of reaction (${\Delta G}_{rxn}$ ) under an applied electrical potential (U ) for steps **1** to **5** as well as **9**, without explicitly accounting for solvated proton‐electron pairs.^[30]^ In the CHE model, we reference zero voltage to the reversible hydrogen electrode (RHE) such that, under standard conditions (298.15 K, 101,325 Pa) and all pH values, a proton and electron pair is in equilibrium with half a molecule of gaseous hydrogen:

(10) $$\left(H^{+}+e^{-}\right)↔\frac{1}{2}H_{2\left(g\right)}$$

and the following relationship between these species’ chemical potentials exists:

(11) $$\mu_{H^{+}}+\mu_{e^{-}}^{U=0}=\frac{1}{2}\mu_{H_{2\left(g\right)}}$$

The chemical potential of $e^{-}$ is linearly related to electrical potential by $\mu_{e^{-}}=\mu_{e^{-}}^{U=0}-eU$ , where e is the elementary positive charge. Thus, we calculate the total chemical potential of a proton‐electron pair under an applied potential as:

(12) $$\mu_{H^{+}}+\mu_{e^{-}}=\frac{1}{2}\mu_{H_{2\left(g\right)}}-eU$$

The free energy of $H_{2\left(g\right)}$ can be readily calculated using standard equations of statistical thermodynamics combined with DFT, thereby allowing us to compute the free energy change for steps **2** to **5** without explicit calculation of the free energy of $H^{+}$ and $e^{-}$ . We calculate the free energy of adsorption ($\Delta G_{ads}$ ) for each state in the ORR pathway with respect to reaction **6**, setting the free energy G of the final state to be 0 eV and that of the initial state to be 4.92 eV, with the latter corresponding to the standard ORR reaction potential from experiment. By doing so, we avoid using O_2(g)_ as a reference species (for which the bond energy is not well‐described using DFT); instead, we use H_2(g)_, H_2_O_(g)_ and the clean slab as references.^[30]^ We note that we calculate the entropic contributions to the free energy at 101,325 Pa and 298.15 K.[^30^, ^31^] Therefore, under acidic conditions (pH=0), $\Delta G_{ads}$ is given as

(13) $$\Delta G_{ads}={\Delta E}_{tot}+{\Delta E}_{ZPE}-T\Delta S-neU$$

where ${\Delta E}_{tot}$ is the change in the DFT total energy, ${\Delta E}_{ZPE}$ is the change in the zero‐point energy correction calculated from harmonic DFT frequencies and ΔS is the change in the entropic contribution from translational, vibrational and rotational motion. More negative values of $\Delta G_{ads}$ correspond to more exergonic adsorption. For each of the ORR steps **1** to **5** we calculate the free energy of reaction for step i (i=2,3,4,5 ) as the difference in the free energy between the final and initial states:

(14) $$\Delta G_{rxn}=\Delta G_{ads}^{Final}-\Delta G_{ads}^{Initial}$$

such that negative values of $\Delta G_{rxn}$ correspond to an exergonic process. Finally, we define the theoretical overpotential (η )^[30]^ as the difference between the equilibrium potential and the potential required to ensure all steps **2** to **5** are thermodynamically downhill. Thus, we express the theoretical overpotential as:

(15) $$\eta =\max_{i}\left(\Delta G_{rxn}^{i}\right)\;for\;U=1.23\;\;V;\;i=2,3,4,5.$$

We use the value of η as an indicator for catalytic performance, whereby lower values of η indicate more effective ORR catalysis.[^17^, ^30^]

### Kinetic Monte Carlo Simulations

We perform simulations within the graph‐theoretical KMC framework as implemented in *Zacros*, version 2.0.[^32^, ^33^, ^34^] Simulations are performed at 298.15 K and the pressure of gas phase CO (p_CO_) is varied from 10^−20^ to 10^0^ atm. The simulation cells consist of rectangular unit cells with 6‐fold symmetry akin to the (111) surface and each lattice has a total of 3240 active metal atoms. For all simulations the surface is initially bare, and we ensure the system reaches steady state before sampling 100 configurations at time intervals of 100 s and calculating the mean CO coverage ($\theta_{CO}$ ). We note that $\theta_{CO}$ is normalised by the number of active metal atoms.

### Rate Constants from Density Functional Theory

Kinetic constants for CO adsorption/desorption events are computed on each surface. According to transition state theory (TST),[^35^, ^36^] the rate constant $k_{TST}$ of an elementary process can be calculated as

(16) $$k_{TST}=\frac{k_{B}T}{h}\cdot \frac{Q^{TS}}{Q^{IS}}exp\left(-\frac{\Delta E_{a}}{k_{B}T}\right)$$

where$\;k_{B}$ is the Boltzmann constant, h is Planck's constant, T is the temperature, $Q^{TS}$ and $Q^{IS}$ are the molecular quasi‐partition functions (not accounting for the potential energy) for the transition state and initial state, respectively, and $\Delta E_{a}\;$ is the activation barrier. The adsorption of CO is non‐activated, so $\Delta E_{a}$ for CO desorption is simply the CO adsorption energy which we take from our previous work in Ref. [18]. Moreover, since CO adsorption is exergonic, the transition state can be taken as the gas‐phase species with “one less” translational degree of freedom (this missing degree of freedom corresponds to the reaction coordinate: the distance from the surface). Thus, equation (16) for CO desorption becomes

(17) $$k_{TST}=\frac{k_{B}T}{h}\cdot \frac{Q_{2Dtrn,rot,vib}^{CO_{\left(g\right)}}}{Q_{vib}^{CO}{}^{*}}\cdot \exp\left(\frac{E_{ads}}{k_{B}T}\right)$$

where $Q_{2Dtrn,\;rot,vib}^{{CO}_{\left(g\right)}}$ contains 2 translational, 2 rotational and 1 vibrational contributions and $Q_{vib}^{CO{}^{*}}$ contains only vibrational contributions. For the vibrational components of these partition functions we use the vibrational frequency data from Ref. [18], under the harmonic approximation.^[37]^ Finally, we note that we do not account for any CO−CO lateral interactions for simulations on SAA lattices as, within the range of p_CO_, host Ag and Au atoms remain unoccupied by CO and dopant atoms are isolated from one another. However, for simulations on Pt(111), we account for first nearest‐neighbour CO−CO lateral interactions and use the DFT setup in Ref. [18] to ensure consistency.

### Data Availability

The DFT data that support the findings of this study are openly available in NoMaD at https://dx.doi.org/10.17172/NOMAD/2021.01.02‐1, dataset ID: 6vYHLnjkQwKJgh7BCVq4Jg. The data includes the structures of the pristine slabs and the adsorbed configurations of O_2_, OOH, OH and O for CoAg, CoAu, NiAg, NiAu, PdAg, PdAu, PtAg, PtAu, RhAg, RhAu, as well as O_2_ dissociation transition states for PdAg, PdAu and PtAg. Also included are the calculations of the supplementary material for the aforementioned pristine slabs and adsorbed configurations with the PBE−D3 functional.

## Results and Discussion

In this section, we present the findings of our DFT study on the ORR behaviour of SAAs. We begin by providing details on the most favourable adsorption geometries of key ORR intermediates O_2_*, OOH*, O* and OH*, comparing and contrasting their adsorption free energies at U= 0 V ($\Delta G_{ads}^{0}$ ). We go on to evaluate the thermodynamic limiting potential on each material and compare the selectivity between two $e^{-}$ and four $e^{-}$ reduction. We then discuss the dissociation of O_2_* on each surface without preconceptions about the most probable pathway for ORR in each case. Finally, using DFT data from our previous studies,^[18]^ we parameterise KMC simulations for the most promising SAAs to determine the equilibrium coverage of CO at various partial pressures and compare this with pure Pt(111). The results enable us to provide estimates of the activity based on the total number of available active sites on each model catalyst and to give an assessment on the overall predicted performance of SAAs for the ORR.

### Adsorption Behaviour of ORR Intermediates

**Figure 1:**
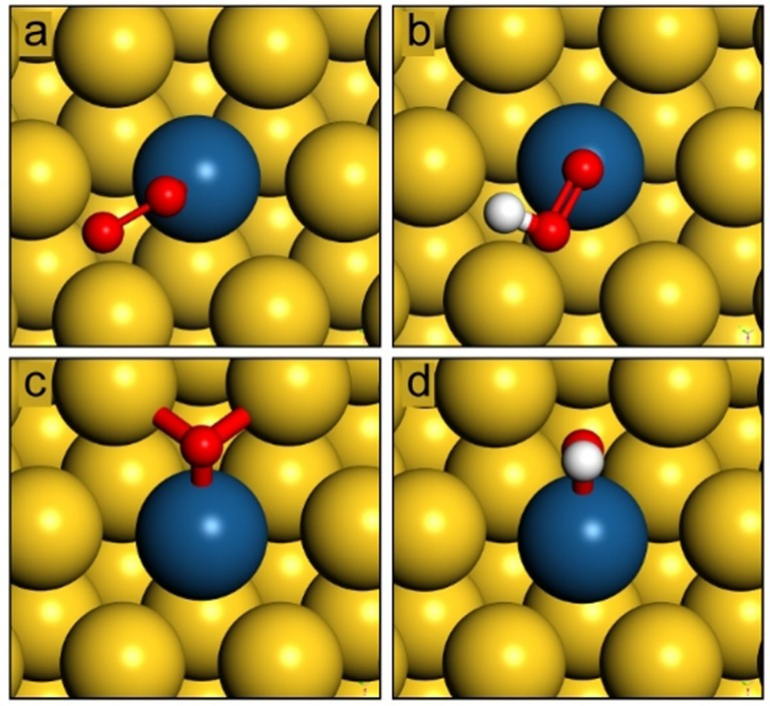
The most favourable adsorption geometries calculated by DFT of a) O_2_*, b) OOH*, c) O* and d) OH* on PtAu(111). The adsorption geometries on PtAu(111) are typical of all SAAs in this study for which geometries can be found in the supporting information.

The first step in the two $e^{-}$ and four $e^{-}$ (both associative and dissociative) pathways is the non‐electrochemical adsorption of molecular O_2_. O_2_* adsorbs weakly to Au(111) and much more strongly to Ag(111), binding in an η_2_ top‐bridge‐top configuration in both cases. From DFT, we calculate $\Delta G_{ads}^{0}$ (O_2_*) to be 4.58 eV and 5.21 eV on Ag and Au, respectively (note that the Gibbs free energy of the left‐hand side of equation 6 is 4.92 eV at U= 0 V). Thus, O_2_* binding is endergonic on Au and is largely responsible for its poor ORR performance. Doping Ag and Au hosts with single atoms of Ni, Pd, Pt, Co and Rh enhances the O_2_* binding strength in all cases with $\Delta G_{ads}^{0}$ (O_2_*) ranging from 3.10 eV to 4.78 eV (Table 1). Thus, for SAAs, O_2_* adsorption is exergonic and therefore doping with single atoms of more reactive metals alleviates the adsorption limitations of monometallic Au ORR catalysts. Regarding adsorption geometry, our DFT calculations reveal that O_2_* binds most favourably over a shared dopant‐host‐host hollow site (Figure 1a) on all SAAs in the study. In this geometry, one O atom bonds primarily to the dopant atom with the other atom coordinated with two 1^st^ nearest neighbour host atoms.

**Table 1:** The free energies of adsorption (U= 0 V) in eV of all ORR intermediates in the four $e^{-}$ pathway on Ag and Au based SAAs, as well as the pure host materials. The free energies of O_2_* adsorption are also given with respect to the O_2 (g)_ formation energy (4.92 eV) in parenthesis.

	$\Delta G_{ads}^{0}$ (eV)			
	O_2_*	OOH*	O*	OH*
CoAg	3.10 (−1.82)	3.27	0.16	−0.18
CoAu	3.66 (−1.26)	3.63	0.80	0.28
NiAg	3.62 (−1.30)	3.47	0.76	0.13
NiAu	4.20 (−0.72)	3.58	1.38	0.56
PdAg	4.34 (−0.58)	3.73	1.67	0.72
PdAu	4.78 (−0.14)	4.03	2.11	1.16
PtAg	4.26 (−0.66)	3.70	1.53	0.81
PtAu	4.67 (−0.25)	3.82	1.91	1.14
RhAg	3.68 (−1.24)	3.26	0.99	0.47
RhAu	4.05 (−0.87)	3.44	1.44	0.85
Ag	4.58 (−0.34)	3.83	1.82	0.74
Au	5.21 (+0.29)	4.40	2.35	1.33

Reduction of O_2_* yielding OOH* is the second step in the two $e^{-}$ and associative four $e^{-}$ ORR pathways. Similarly to O_2_*, OOH* binds weakly on Au and notably stronger on Ag with $\Delta G_{ads}^{0}$ (OOH*) of 4.40 eV and 3.83 eV, respectively. On SAAs, the binding strength is enhanced by the dopant atom with $\Delta G_{ads}^{0}$ (OOH*) ranging from 3.26 eV to 4.03 eV (Table 1). OOH* primarily bonds to SAAs through the non‐protonated O atom interacting with the dopant atom. The protonated O atom binds to just one surface host atom with the O−O bond axis aligned over a shared dopant‐host bridge site. The OOH* adsorbate is slightly tilted and thus not parallel to the surface (Figure 1b).

O* is an intermediate found in the associative and dissociative four $e^{-}$ ORR pathways, arising as a result of OOH* reduction or O_2_* bond scission, respectively. O* is subsequently reduced to OH* and ultimately to water. O* and OH* bind most favourably to fcc hollow and bridge sites, respectively on Ag and Au; however, on SAAs both bind in shared dopant‐host‐host sites (Figure 1c). Ag and Au bind O* in fcc hollow sites with $\Delta G_{ads}^{0}$ (O*) of 1.82 eV and 2.35 eV, respectively, as well as OH* in bridge sites with $\Delta G_{ads}^{0}$ (OH*) of 0.74 eV and 1.33 eV, respectively. On SAAs the binding strengths of both species are enhanced due to doping, compared to the pure host materials, with $\Delta G_{ads}^{0}$ (O*) ranging from 0.16 eV to 2.11 eV and $\Delta G_{ads}^{0}$ (OH*) ranging from −0.18 eV to 1.16 eV (Table 1).

**Figure 2:**
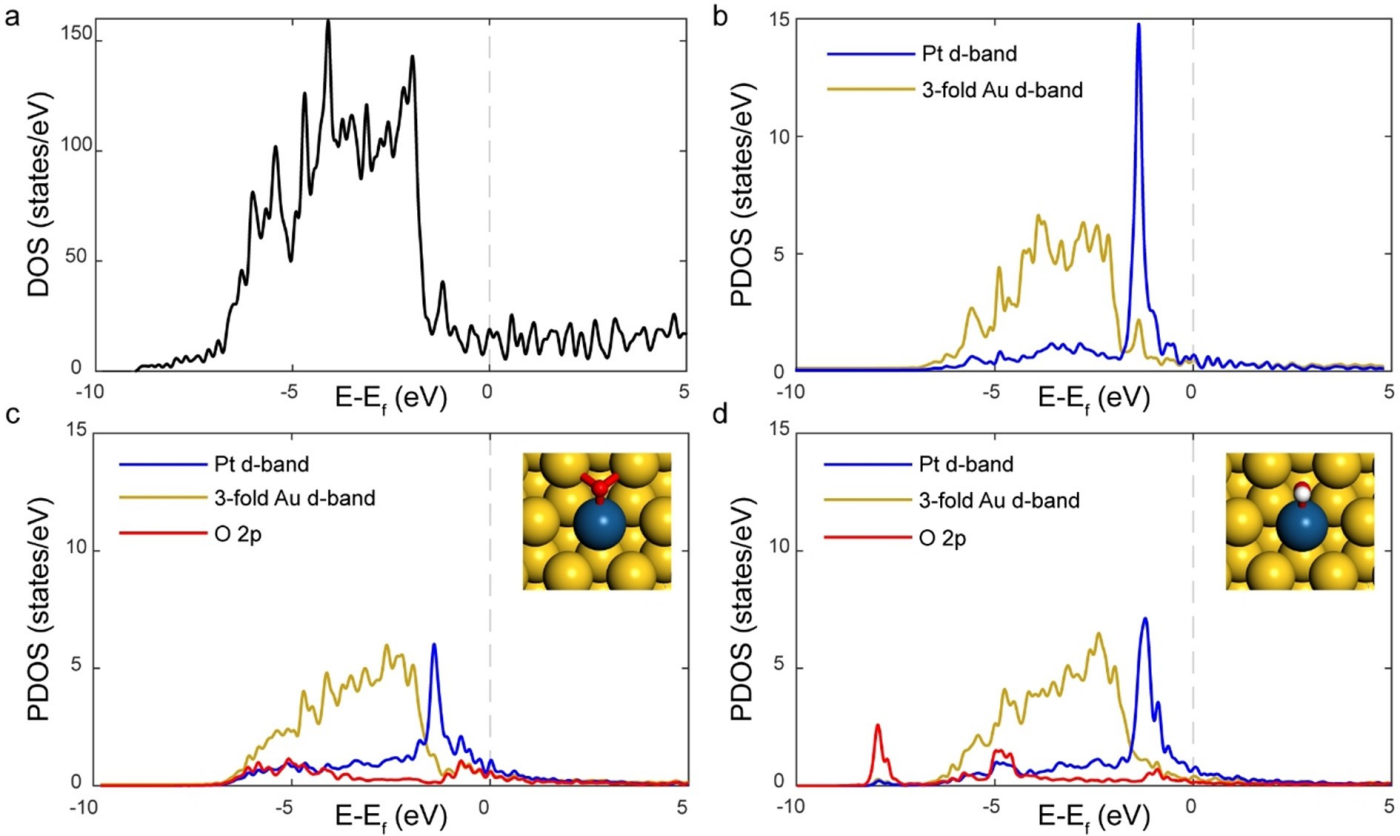
Electronic DOS plots for the PtAu system showing a) the total DOS; b) PDOS for the Pt dopant atom d‐band (blue) and summed d‐band contributions of the two surface Au atoms forming a shared Pt−Au−Au fcc hollow site (gold); c) PDOS for that in (b) after O* adsorption (inset) as well as the O 2p band (red); and d) PDOS for that in (b) after OH* adsorption (inset) as well as the O 2p band.

In general, we find that Ag‐based SAAs bind ORR intermediates more strongly than their Au counterparts by approximately 0.4 to 0.6 eV (Table 1). This is consistent with the higher reactivity of pure Ag towards ORR species compared to pure Au. Analysis of the projected density of states (PDOS) of Ag‐ and Au‐based SAAs by Thirumalai and Kitchin, demonstrates that the dopant atom behaves like a free atom due to ineffective mixing with the host material electron density.^[38]^ Analysing the electronic structure of a representative SAA, PtAu (Figure 2), we note a sharp feature close to the Fermi level (Figure 2b) which we attribute to the d‐band of the isolated Pt atom, in good agreement with Thirumalai and Kitchin. This property is thought to be responsible for the excellent surface reactivity exhibited by SAAs.^[38]^ Since the sharp peak in the dopant atom d‐band electron density is close to the Fermi level (Figure 2b), it contributes a greater amount of electron density to the metal‐adsorbate hybrid bands than the lower lying d‐band of the host material, thereby enhancing the reactivity of the latter. Thirumalai and Kitchin revealed that Ag host atoms have less electron density mixing with dopant atoms than Au does, which is evidenced by sharper dopant atom d‐band peaks when embedded in the former over the latter.^[38]^ Moreover, the dopant d‐band peaks are shifted closer to the Fermi level for Ag‐based SAAs compared to Au‐based SAAs and are thus more reactive,^[38]^ as our calculations also demonstrate here. Generally, the dopant that exhibits the strongest binding is Co, with Ni and Rh exhibiting weaker but still moderate binding. Pd and Pt exhibit the weakest binding strength, though it is still enhanced compared to pure Ag and significantly higher compared with Au.

**Figure 3:**
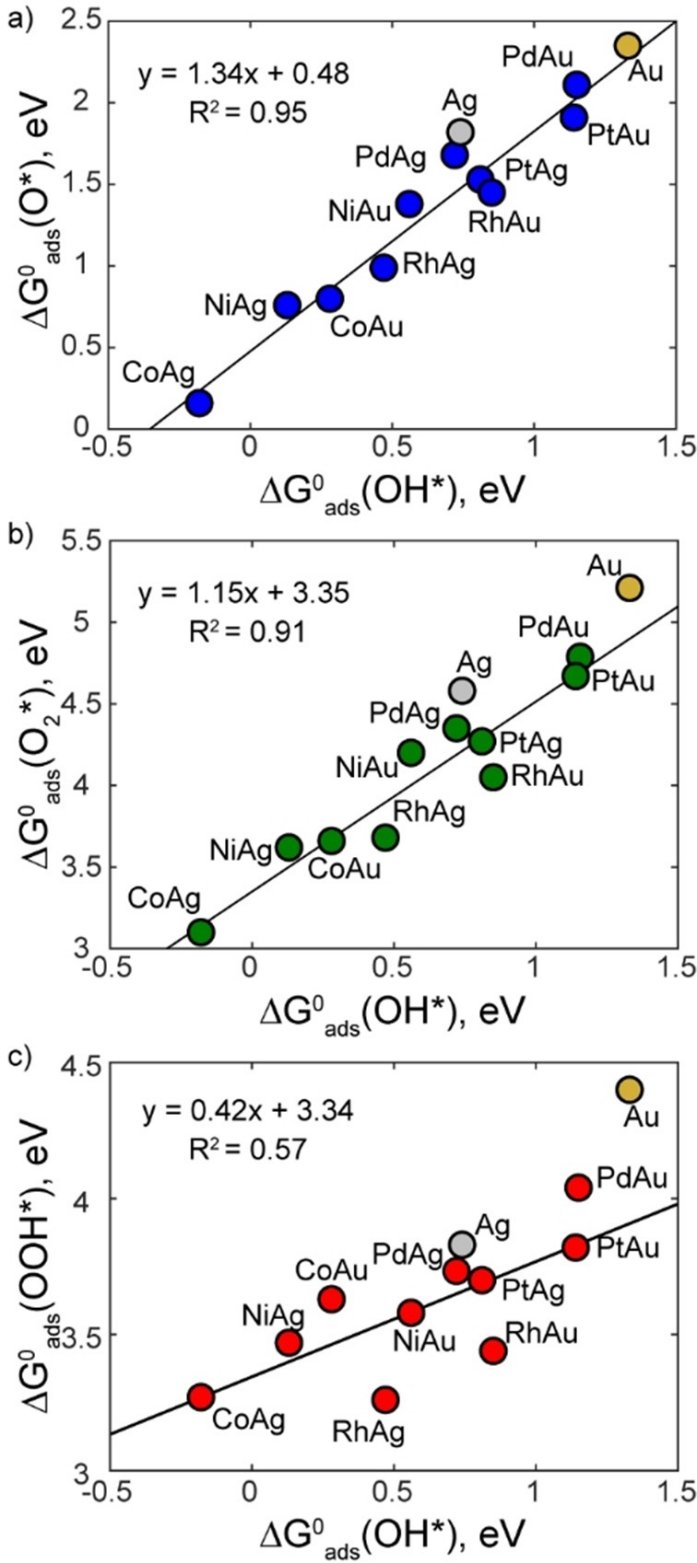
Thermochemical linear scaling relations between the free energy of adsorption for OH* and a) O* (blue), b) O_2_* (green) and c) OOH* (red). Linear regression lines are plotted for the SAA data only. Ag and Au data points are marked for reference but are not included in the fitting.

Interestingly when we consider trends in the values of $\Delta G_{ads}^{0}$ for different intermediates across the SAA surfaces, we determine a strong positive linear correlation between $\Delta G_{ads}^{0}$ (O*) and $\Delta G_{ads}^{0}$ (OH*), as well as a good correlation between $\Delta G_{ads}^{0}$ (O_2_*) and $\Delta G_{ads}^{0}$ (OH*) (Figure 3). However, the correlation between $\Delta G_{ads}^{0}$ (OOH*) and $\Delta G_{ads}^{0}$ (OH*) is notably weaker (R^2^=0.57). Most materials studied for ORR behaviour follow “standard” scaling relations with well‐correlated $\Delta G_{ads}^{0}$ for these species.^[17]^ OH* and OOH* generally bond to most materials via one O atom which forms a single bond with the surface, giving rise a scaling slope close to unity. On the other hand, O* binds with a formal double bond compared to the OH* single bond giving rise to a slope of around two.^[17]^ In the case of SAAs there is a strong scaling relation between $\Delta G_{ads}^{0}$ (OH*) and $\Delta G_{ads}^{0}$ (O*) whereas for $\Delta G_{ads}^{0}$ (OH*) vs. $\Delta G_{ads}^{0}$ (OOH*) the correlation is poor.

We have shown in the past that SAAs may not always follow the traditional linear scaling relations of other metals and their alloys; instead, they are observed to either exhibit distinct linear trends of their own or deviating entirely from linearity.^[16]^ The implication of this behaviour is that SAAs often exhibit unique catalytic properties that can create novel opportunities in rational catalyst design, as discussed in the perspective in Ref. [11]. We previously attributed this to the surface inhomogeneity of SAAs combined with adsorbate site valency rules.^[16]^ For example O* generally binds to (111) surfaces in threefold hollow sites (as is the case here), whereas higher‐valent OOH* fragments will bind on top or, in this case, bridge sites. The consequence for binding this way on SAAs, is that O* interacts with one surface dopant atom and two surface host atoms, whereas OOH* interacts with one dopant atom but only one host atom. This decouples the binding strength of these fragments and we determine weakly correlated scaling relations. Similarly for O* and OH*, they require two and one electron(s), respectively, to fill their octet. Since both species are bound to the same fcc hollow site, they have bonding contributions from the same number of surface host and dopant atoms, therefore their adsorption free energies scale well. This is shown in Figure 2c–d where the PDOS shows the surface‐adsorbate bonding interaction as having contributions from both the Au and Pt d‐bands. In addition, in the case of O* (Figure 2c), the O 2p band is much broader than for OH* (Figure 2d) due to the formation of two formal adsorbate‐surface bonds instead of one, the relative contribution to the bonding from Pt and Au is comparable in both cases.

### Potential Dependent Free Energy Analysis

We have analysed the thermodynamics of the ORR pathways on each SAA to compare the selectivity between the two $e^{-}$ and four $e^{-}$ reduction pathways (i. e. H_2_O_2_ vs. H_2_O production), as well as to identify the most promising materials that exhibit the lowest theoretical overpotential. η is the maximum additional potential above the ORR equilibrium (1.23 eV) for which every proton‐electron pair reduction step is thermodynamically downhill. When the latter condition is satisfied, the proton transfer barriers are expected to be low and consistent. Thus, the activity of ORR catalysts is often qualitatively predicted based on thermodynamic η , with lower values of η corresponding to greater predicted activity.

**Figure 4:**
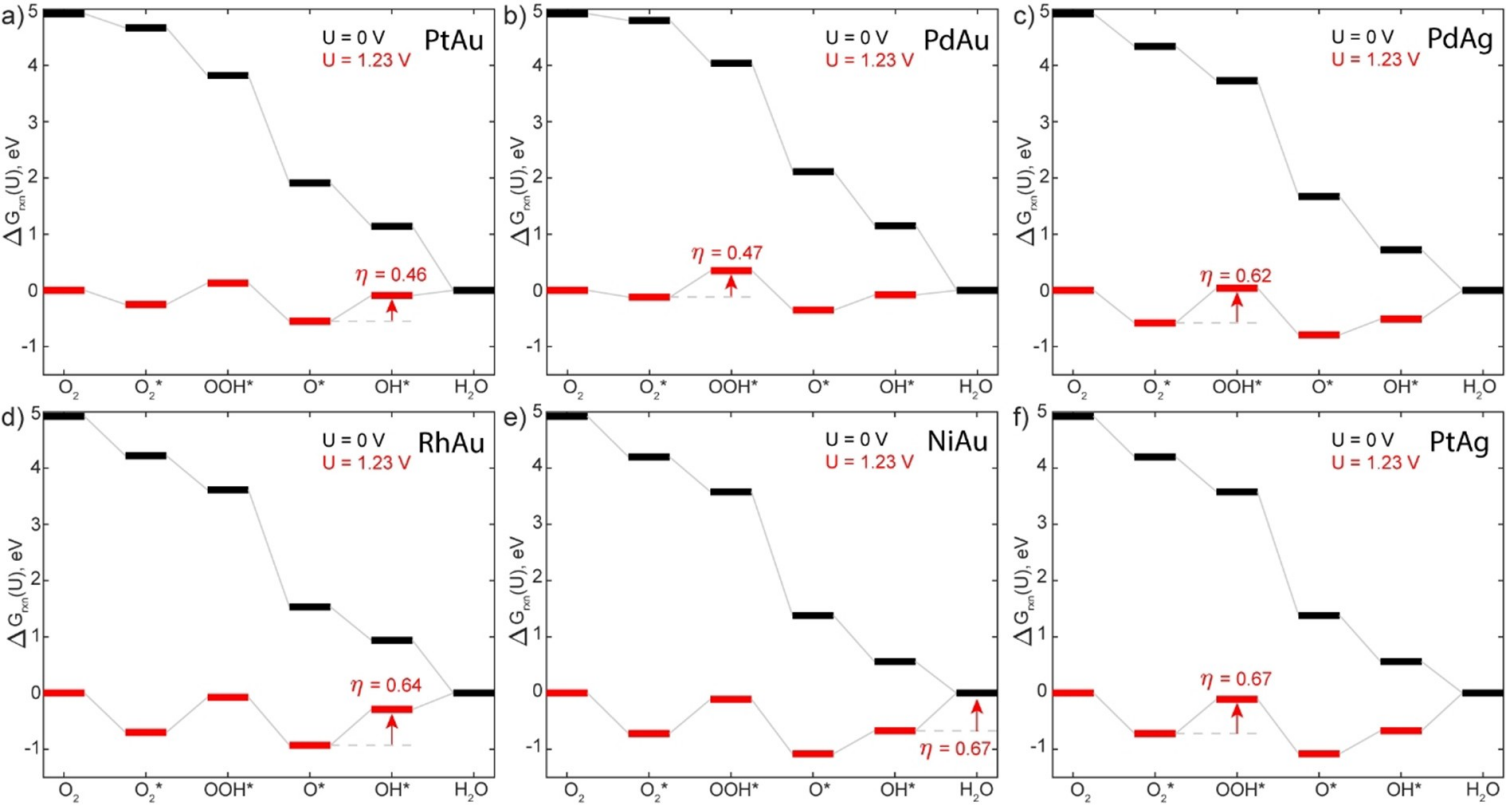
Gibbs free energy profiles for the four $e^{-}$ ORR pathway on the six lowest overpotential SAAs a) PtAu, b) PdAu, c) PdAg, d) RhAu, e) NiAu and f) PtAg.

Our results indicate that SAAs exhibit values of η ranging from 0.46 eV to 1.41 eV (Table 2), where η (PtAu) <η (PdAu) <η (PdAg) <η (RhAu) <η (NiAu) ≈η (PtAg) <η (RhAg) <η (NiAg) <η (CoAu) <η (CoAg), with the top six (i. e. those with the lower η ) being shown in Figure 4 (the values of η (NiAu) and η (PtAg) are 0.672 eV and 0.669 eV, respectively, up to 3 decimals). Interestingly, there is a general trend where SAAs with a Au host matrix outperform their Ag counterparts. This trend is consistent with Ag‐based SAAs binding ORR intermediates more strongly than their Au counterparts, which results in greater free energy differences along the reaction coordinate. The greater binding strength of Ag‐based SAAs is attributed to sharper dopant d‐band peaks in the PDOS that are closer to the Fermi level compared to their Au‐based counterparts, as this facilitates more effective hybridisation and filling of surface‐adsorbate bands.^[38]^

**Table 2:** The free energy of reactions in eV for steps 1–5 in the four e^−^ ORR pathway at *U*=1.23 V, as well as the corresponding theoretical overpotential.

SAA	$\Delta G_{rxn}^{1}$	$\Delta G_{rxn}^{2}$	$\Delta G_{rxn}^{3}$	$\Delta G_{rxn}^{4}$	$\Delta G_{rxn}^{5}$	η
CoAg	−1.82	1.40	−1.87	0.89	1.41	1.41
CoAu	−1.26	1.21	−1.60	0.71	0.95	1.21
NiAg	−1.30	1.08	−1.47	0.59	1.10	1.10
NiAu	−0.72	0.61	−0.97	0.41	0.67	0.67
PdAg	−0.58	0.62	−0.83	0.28	0.51	0.62
PdAu	−0.14	0.47	−0.69	0.28	0.07	0.47
PtAg	−0.66	0.67	−0.94	0.51	0.42	0.67
PtAu	−0.25	0.38	−0.68	0.46	0.09	0.46
RhAg	−1.24	0.81	−1.03	0.71	0.76	0.81
RhAu	−0.87	0.62	−0.77	0.64	0.38	0.64

The best performing SAAs are PtAu and PdAu with η values of 0.46 eV and 0.47 eV. Contextualising these values of η , Pt(111) (the DFT model surface for the Pt/C electrode) has a value of 0.48 eV.^[17]^ Thus, our calculations predict that the dopant atoms of PtAu and PdAu SAAs will exhibit comparable ORR kinetics over individual atoms within Pt(111). Of course, the total number of active Pt atoms within Pt(111) is substantially greater (20‐ to 100‐fold) than those in a SAA surface and therefore despite a comparable η , the activity of the Pt/C electrode for ORR will likely be much greater than the best performing SAAs. We also performed these calculations with a D3 dispersion correction (PBE−D3) and found that the O_2_* binding strength is increased. This leads to small changes in η , however the overall trends across the SAAs remain consistent (see supporting information). However, there may still be advantages of using SAAs over traditional Pt electrodes, such as high selectivity and tolerance to poisoning, which we discuss in subsequent sections.

### Dissociation of O_2_ Dimer

The key difference between the associative and dissociative four $e^{-}$ pathways is that the O−O bond in O_2_* is cleaved in the latter, bypassing the formation of the OOH* intermediate and the mitigating the risk of forming H_2_O_2_ as a product. Instead, two O* adatoms are formed, and each is reduced twice to yield H_2_O via OH*. Of the 10 SAAs studied here, O_2_* reduction to OOH* is the thermodynamically limiting step for five materials (CoAu, PdAg, PdAu, PtAg and RhAg). Thus, these five SAAs may stand to benefit thermodynamically from the O_2_ scission step, since this will bypass OOH* formation and subsequent protonation. Studying the potential‐dependent reaction free energies for each of the five SAAs (Table 2), we note that η is only reduced to values indicating high activity for the dissociative vs. associative pathway for PdAu, PdAg and PtAg. Indeed, if OOH* formation was bypassed on CoAu and RhAg, the next thermodynamically limiting step in both cases is reaction 5 (reduction of OH* to H_2_O) for which $\Delta G_{rxn}^{5}=\;$ 0.95 eV and 0.76 eV, respectively. On the other hand, the subsequent limiting step for PdAu is reaction 4 (reduction of O* to OH*, $\Delta G_{rxn}^{4}=$ 0.28 eV), for PdAg reaction 5 ($\Delta G_{rxn}^{5}=\;$ 0.51 eV), and for PtAg reaction 4 ($\Delta G_{rxn}^{4}=$ 0.51 eV). The pertinent $\Delta G_{rxn}$ values are sufficiently low that they suggest fast kinetics. We therefore compute the O_2_* dissociation barrier for these materials to determine which pathway will be most feasible under ambient conditions.

Our calculations of the O_2_* dissociation transition states yield activation barriers of 0.71 eV for PdAg, 0.86 eV for PdAu and 0.77 eV for PtAg. Of these three SAAs, no material exhibits an O_2_* dissociation barrier that is lower than η suggesting that they will not facilitate ORR via the dissociative mechanism and bypass OOH* formation. Though the barriers for PdAg and PtAg are greater than the η that is dictated by the O_2_* reduction step, they are similar. Given the reduction of O_2_* to OOH* is unlikely to be a barrierless process, if we considered a small, approximate activation energy (i. e. 0 to 0.1 eV) then the kinetics of O_2_* dissociation and reduction would appear to be comparable. Thus, we predict that the kinetics of the associative and dissociative four $e^{-}$ pathways on PdAg and PtAg will be competitive, close to the equilibrium potential. At the equilibrium potential, the activation barrier for O_2_* dissociation on PdAu is 0.39 eV greater than $\Delta G_{rxn}^{2}$ for the rate limiting OOH* formation step and we suppose this to be sufficiently high for O_2_* reduction and the associative pathway to be dominant. Indeed for more negative potentials (i. e. approaching the limiting potential), the activation barrier will become increasingly greater than the thermodynamic limiting step and therefore the associative pathway will be more dominant.

### Four Electron vs. Two Electron Selectivity

A common issue with Pt‐based ORR catalysts is selectivity towards water via four e^−^reduction relative to hydrogen peroxide via the two $e^{-}$ pathway. ORR to water through the four $e^{-}$ pathway is more efficient, as the two $e^{-}$ pathway involves incomplete oxygen reduction that results in low energy conversion efficiency, as well as the formation of unwanted intermediate and radical species. On Pt, site blocking by H* adatoms combined with comparable free energies around the operating potential of OOH* reduction to H_2_O_2_ and OH* reduction to H_2_O mean that the two $e^{-}$ pathway is prevalent. Thus, we assess the thermodynamic selectivity of the four versus the two $e^{-}$ pathways on SAAs.

We use the values of the thermodynamically limiting potential ($U_{T}$ ) determined in the previous section to determine the free energy landscape for each SAA operating at their most efficient potential (i. e. the lowest overpotential). At $U_{T}$ , by definition all steps in the four $e^{-}$ pathway are thermodynamically downhill. We assess the selectivity by comparing the difference in free energy of reaction at $U_{T}$ for the OOH* reduction step to H_2_O_2_ ($\Delta G_{rxn}^{H2O2}\left(U_{T}\right)$ ) and for the thermodynamically limiting step along the four $e^{-}$ pathway after the formation of OOH* ($\Delta G_{rxn}^{i}\left(U_{T}\right),\;i=3,4,5$ ) such that:

(18) $$\Delta \Delta G_{sel}\left(U_{T}\right)=\Delta G_{rxn}^{H2O2}\left(U_{T}\right)-\max_{i}\left(\Delta G_{rxn}^{i}\left(U_{T}\right)\right)$$

If $\Delta \Delta G_{sel}\left(U_{T}\right)$ is positive, there is a thermodynamic selectivity for the four $e^{-}$ over two $e^{-}$ reduction at $U_{T}$ and the more positive the value, the greater the thermodynamic selectivity.

In all cases, $\Delta \Delta G_{sel}\left(U_{T}\right)$ is positive with values ranging from the least selective at 0.16 eV to the most selective at 0.82 eV (Table 3), with the latter being a considerable improvement over Pt for which $\Delta \Delta G_{sel}\left(U_{T}\right)$ =0.35 eV.^[39]^ In general, the Co‐doped SAAs are the least selective and happen to be the ones that bind ORR intermediates the strongest. The majority of the other SAAs have a value of $\Delta \Delta G_{sel}\left(U_{T}\right)$ between 0.5 and 0.6 eV indicating fairly strong selectivity towards four $e^{-}$ reduction. Finally, the Rh‐doped SAAs exhibit the greatest selectivity with $\Delta \Delta G_{sel}\left(U_{T}\right)$ close to 0.8 eV. For SAAs exhibiting the lowest η , namely PtAu and PdAu, the selectivity is reasonably strong with $\Delta \Delta G_{sel}\left(U_{T}\right)$ of 0.56 eV and 0.54 eV, respectively. Thus PdAu and PtAu SAAs exhibit enhanced selectivity over pure Pt, with values of $\Delta \Delta G_{sel}\left(U_{T}\right)$ that, compared to the latter, favour four over two $e^{-}$ reduction by an additional ∼0.2 eV.

**Table 3:** Reaction free energies at the thermodynamically limiting potential $U_{T}$ for possible reduction steps following the formation of OOH* including for the final step in the two $e^{-}$ ORR pathway $\Delta G_{rxn}^{H2O2}\left(U_{T}\right)$ and the remaining three steps in the four $e^{-}$ pathway ($\Delta G_{rxn}^{i}\left(U_{T}\right),\;i=3,4,5$ ). The thermodynamic selectivity is given by $\Delta \Delta G_{sel}\left(U_{T}\right)$ .

SAA	$\Delta G_{rxn}^{H2O2}\left(U_{T}\right)$	$\Delta G_{rxn}^{3}\left(U_{T}\right)$	$\Delta G_{rxn}^{4}\left(U_{T}\right)$	$\Delta G_{rxn}^{5}\left(U_{T}\right)$	$\Delta \Delta G_{sel}\left(U_{T}\right)$
CoAg	0.16	−3.28	−0.52	0.00	0.16
CoAu	−0.01	−2.81	−0.50	−0.26	0.25
NiAg	0.26	−2.58	−0.51	0.00	0.26
NiAu	0.59	−1.64	−0.26	0.00	0.59
PdAg	0.49	−1.44	−0.34	−0.10	0.60
PdAu	0.34	−1.16	−0.20	−0.40	0.54
PtAg	0.47	−1.61	−0.16	−0.25	0.63
PtAu	0.56	−1.14	0.00	−0.37	0.56
RhAg	0.77	−1.84	−0.10	−0.05	0.82
RhAu	0.76	−1.40	0.00	−0.26	0.76

### Evaluating CO Tolerance

Our calculations so far have revealed that PtAu and PdAu SAAs exhibit theoretical overpotentials that are comparable with Pt(111), as well as strong selectivity for four $e^{-}$ reduction. However, the overall catalytic activity of these SAAs will be significantly lower than the Pt/C electrode, simply because of the low number of reactive sites on the former compared to the latter. Indeed, the density of reactive metal atoms in the surface layer of a SAA is typically around 3–5 %, and thus the number of active sites is significantly reduced compared to pure Pt.[^40^, ^41^, ^42^] However, Pt catalysts are highly susceptible to catalytic poisoning by CO, which is a common impurity in hydrogen gas streams that are utilised in PEM fuel cells. On the other hand, SAAs exhibit strong resistance to poisoning that can be attributed to weak binding of CO.[^15^, ^18^]

In our previous work, we simulated the temperature programmed desorption kinetics of CO from SAAs/PGMs and benchmarked the results against experiments.^[18]^ Our results were in excellent agreement with the available experimental data and the mean absolute error in the KMC‐simulated CO desorption peak temperature was just 13 K.^[18]^ From this study, we calculated CO adsorption energies of −1.48 eV for Pt(111), −1.37 eV for PtAu(111) and −0.88 eV for PdAu(111). Thus for PdAu(111) there is a significant reduction in the affinity for CO binding compared to pure Pt(111), and under temperature programmed desorption conditions we simulated a much lower CO desorption temperature for the former, as also observed experimentally.[^18^, ^43^] Unlike the PdAu SAA, PtAu(111) only exhibits a slight reduction in the CO binding affinity compared to Pt(111) and is unlikely to have significantly enhanced CO tolerance.

**Figure 5:**
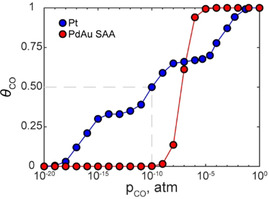
Langmuir isotherm for the KMC simulated adsorption of CO at 298.15 K on Pt(111) (blue) and PdAu(111) SAA (red). The coverage $\theta_{CO}$ is the number of CO molecules normalised by the number of active sites (i. e. number of Pt surface atoms in Pt(111) or Pd atoms in the PdAu(111) SAA). The half‐maximum $\theta_{CO}$ on Pt(111) is indicated by the grey dashed line.

In order to illustrate the extent of CO poisoning resistance on SAAs compared to Pt(111), we use the CO adsorption energies on Pt(111), PtAu(111) and PdAu(111) from Ref. [18] and compute $k_{TST}$ at 298.15 K for use in our KMC model here. Using KMC, we determine $\theta_{CO}$ across a range of $p_{CO}$ and plot the resulting isotherms for Pt(111) and PdAu(111) in Figure 5 (note that $\theta_{CO}$ for PdAu(111) is normalised with respect to the number of Pd active sites). Analysing the isotherms, we observe that Pt(111) begins to poison at $p_{CO}$ of 10^−19^ atm and is saturated by 10^0^ atm. The Pt(111) isotherm also shows the formation of two CO adlayer phases, in excellent agreement with experiment.^[44]^ Moreover, the half‐maximum $\theta_{CO}$ on Pt(111) from experiment is obtained at approximately 6×10^−10^ atm and agrees very well with our simulated $p_{CO}$ of 10^−10^ atm.^[44]^ Considering the PdAu(111) SAA isotherm we simulate a narrow range over which the surface poisons, beginning at $p_{CO}$ of 10^−9^ atm leading to full saturation at 10^−4^ atm. Notably PdAu(111) SAA does not have ordered phases of CO on the surface due to the dispersion of the Pd dopant atoms negating any potential adlayer interaction effects.

Comparing the isotherms, there is approximately 10 orders of magnitude difference in $p_{CO}$ that corresponds to the onset of CO poisoning between Pt(111) and PdAu(111) SAA. Moreover, at the half‐maximum $\theta_{CO}$ point on Pt(111), PdAu(111) SAA remains pristine, indicating a significant increase in the CO tolerance of the latter over the former. Thus, Pt(111) active sites are much more likely to undergo poisoning than Pd sites in PdAu, which ultimately could result in comparable availability of active sites for both and therefore similar activities of these materials despite a 20–100 fold dopant atom dilution in the SAA.

## Conclusions

In this contribution we have systematically studied the behaviour of 10 SAAs towards ORR intermediates. We have elucidated the most favourable adsorption configurations of O_2_*, OOH*, O* and OH* on each SAA, demonstrating that isolated atoms of Ni, Pd, Pt, Co and Rh within the surface matrix of Au(111) and Ag(111) significantly increase the reactivity of their host metal. In general, we found that ORR intermediates bind most strongly to Co SAAs, closely followed by Ni and Rh, with Pd and Pt binding the weakest. Additionally, we determined that Ag based SAAs are more reactive towards ORR intermediates than their Au counterparts. Using the CHE model, we evaluated the potential dependence of ORR thermodynamics on SAAs in order to elucidate the selectivity of four $e^{-}$ over two $e^{-}$ reduction, as well as to yield η as an activity metric. Our results indicate that SAAs, especially those with the lowest overpotentials, have significant thermodynamic bias for water formation via four $e^{-}$ reduction over hydrogen peroxide formation via two $e^{-}$ reduction, indicating they will be highly selective. Evaluation of η , the maximum potential difference from equilibrium at which all steps are thermodynamically downhill and a strong metric for ORR activity, predicts that the best performing SAAs are PtAu and PdAu with overpotentials that are slightly lower than model Pt(111). In view of these calculations the SAAs just noted will be as active as Pt(111) on an individual platinum group metal basis, though one may argue that the low density of these atoms in a SAA (typically 3–5 % of surface atoms) will hamper the observed (apparent) activity. However, we demonstrate that a significant advantage of the PdAu SAA is its resistance to CO poisoning. Crucially, KMC simulations reveal that PdAu is tolerant to CO poisoning for partial pressures that are 10 orders of magnitude greater than for pure Pt, suggesting that during operation, the availability of active sites on each surface will be similar. Therefore, the predicted activity and selectivity of SAAs, along with the high tolerance to CO poisoning, constitute an exciting combination of desirable properties, which makes SAAs attractive for electrocatalytic ORR compared to the traditional Pt/C catalysts. Our theoretical investigations are thus expected to stimulate further research and pave the way towards the experimental synthesis and testing of these promising materials.

## Supporting Information

Comparison of the potential Gibbs free energy diagrams obtained with PBE versus PBE−D3 for the four e‐ ORR on SAAs of Co, Ni, Pd, Pt and Rh doped into Ag and Au hosts.

The DFT data of this paper can be accessed via: https://dx.doi.org/10.17172/NOMAD/2021.01.02‐1

## Conflict of interest

The authors declare no conflict of interest.

## Supporting information

As a service to our authors and readers, this journal provides supporting information supplied by the authors. Such materials are peer reviewed and may be re‐organized for online delivery, but are not copy‐edited or typeset. Technical support issues arising from supporting information (other than missing files) should be addressed to the authors.

Supplementary
