# Supplementary material for: Single‐Atom Alloys for the Electrochemical Oxygen Reduction Reaction
Source: Chemphyschem. 2021 Feb 3;22(5):499–508. doi: 10.1002/cphc.202000869 (PMC7986805; doi:10.1002/cphc.202000869)
Supplement: Supplementary file 1 — Supplementary [file CPHC-22-499-s001.pdf]

ChemPhysChem

Supporting Information

Single-Atom Alloys for the Electrochemical Oxygen Reduction Reaction

Matthew T. Darby and Michail Stamatakis*

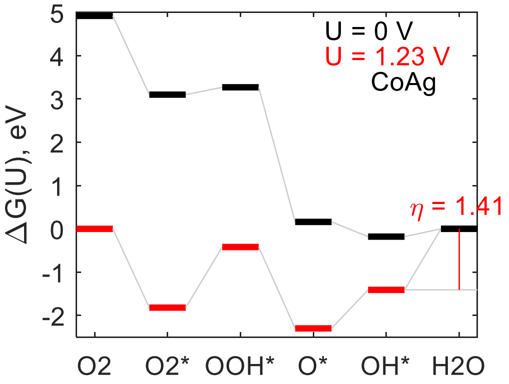
U = 0 V
U = 1.23 V
CoAg
η = 1.41
ΔG(U), eV
5
4
3
2
1
0
-1
-2
O2
O2*
OOH*
O*
OH*
H2O

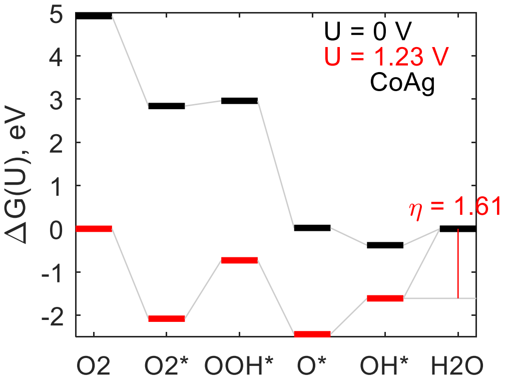
U = 0 V
U = 1.23 V
CoAg
η = 1.61
ΔG(U), eV
5
4
3
2
1
0
-1
-2
O2
O2*
OOH*
O*
OH*
H2O

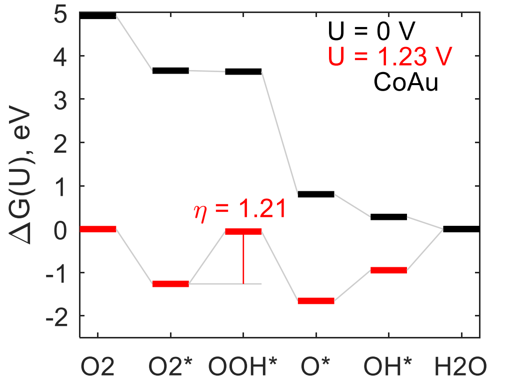
U = 0 V
U = 1.23 V
CoAu
η = 1.21
ΔG(U), eV
5
4
3
2
1
0
-1
-2
O2
O2*
OOH*
O*
OH*
H2O

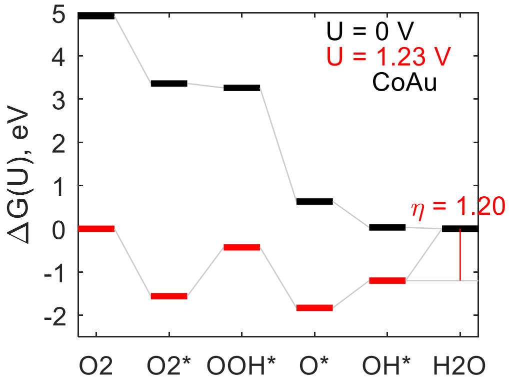
U = 0 V
U = 1.23 V
CoAu
η = 1.20
ΔG(U), eV
5
4
3
2
1
0
-1
-2
O2
O2*
OOH*
O*
OH*
H2O

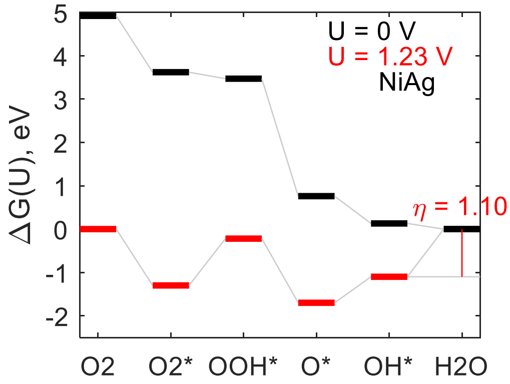
U = 0 V
U = 1.23 V
NiAg
η = 1.10
ΔG(U), eV
5
4
3
2
1
0
-1
-2
O2
O2*
OOH*
O*
OH*
H2O

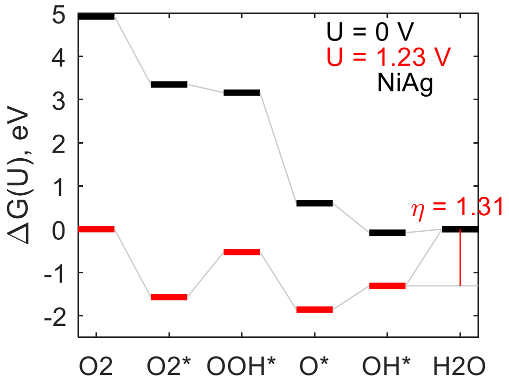
U = 0 V
U = 1.23 V
NiAg
η = 1.31
ΔG(U), eV
5
4
3
2
1
0
-1
-2
O2
O2*
OOH*
O*
OH*
H2O

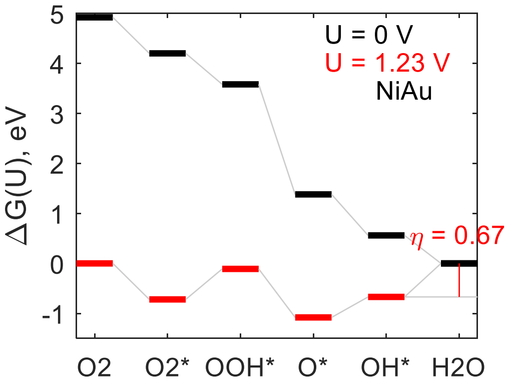
U = 0 V
U = 1.23 V
NiAu
η = 0.67
ΔG(U), eV
5
4
3
2
1
0
-1
O2
O2*
OOH*
O*
OH*
H2O

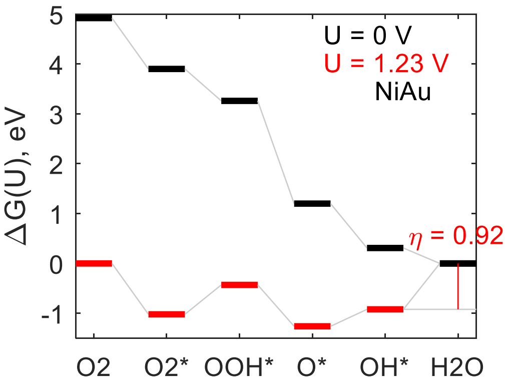
U = 0 V
U = 1.23 V
NiAu
η = 0.92
ΔG(U), eV
5
4
3
2
1
0
-1
O2
O2*
OOH*
O*
OH*
H2O

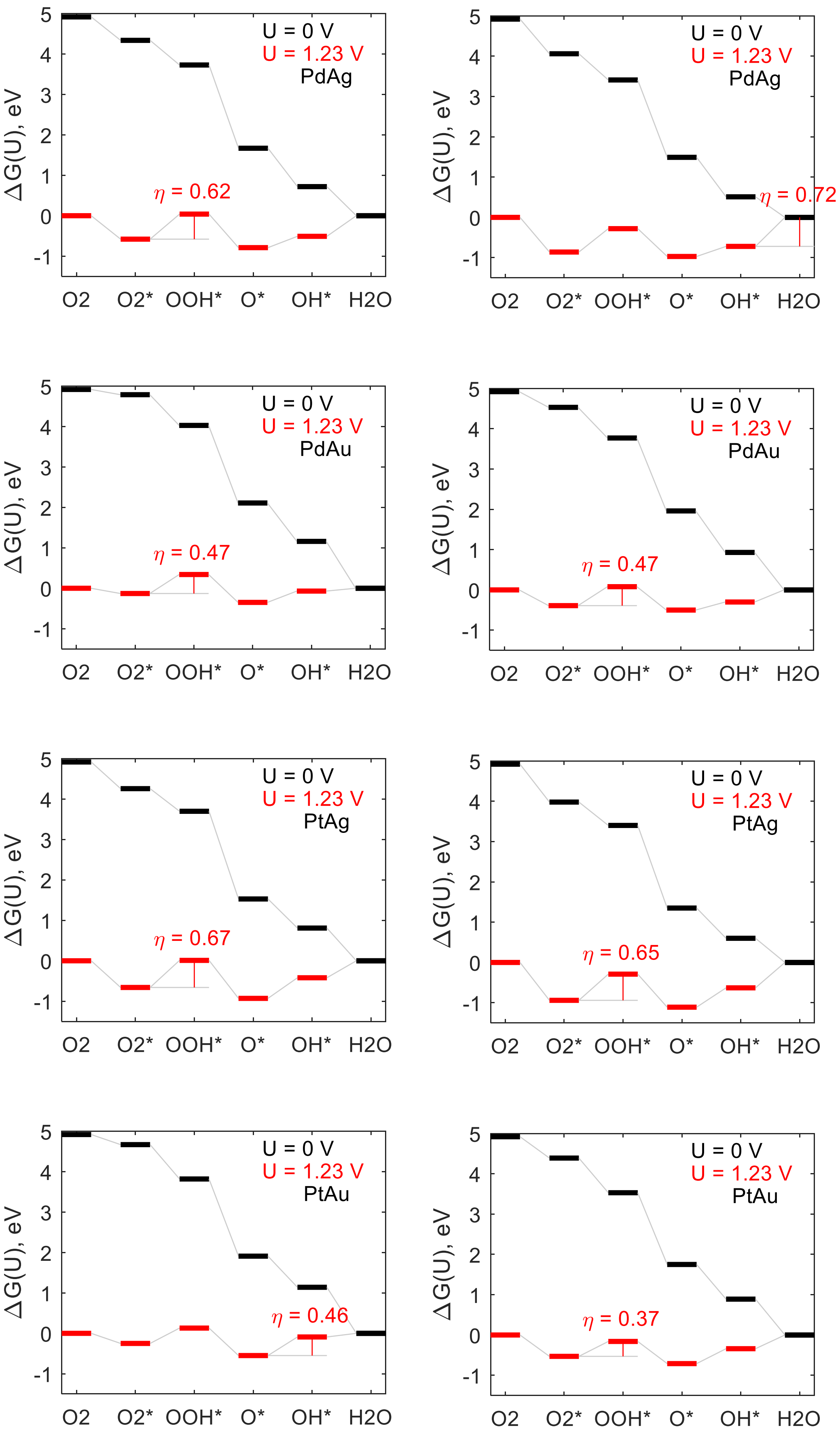

ΔG(U), eV
U = 0 V
U = 1.23 V
PdAg
η = 0.62
PdAg
η = 0.72
PdAu
η = 0.47
PdAu
η = 0.47
PtAg
η = 0.67
PtAg
η = 0.65
PtAu
η = 0.46
PtAu
η = 0.37
O2
O2*
OOH*
O*
OH*
H2O
5
4
3
2
1
0
-1

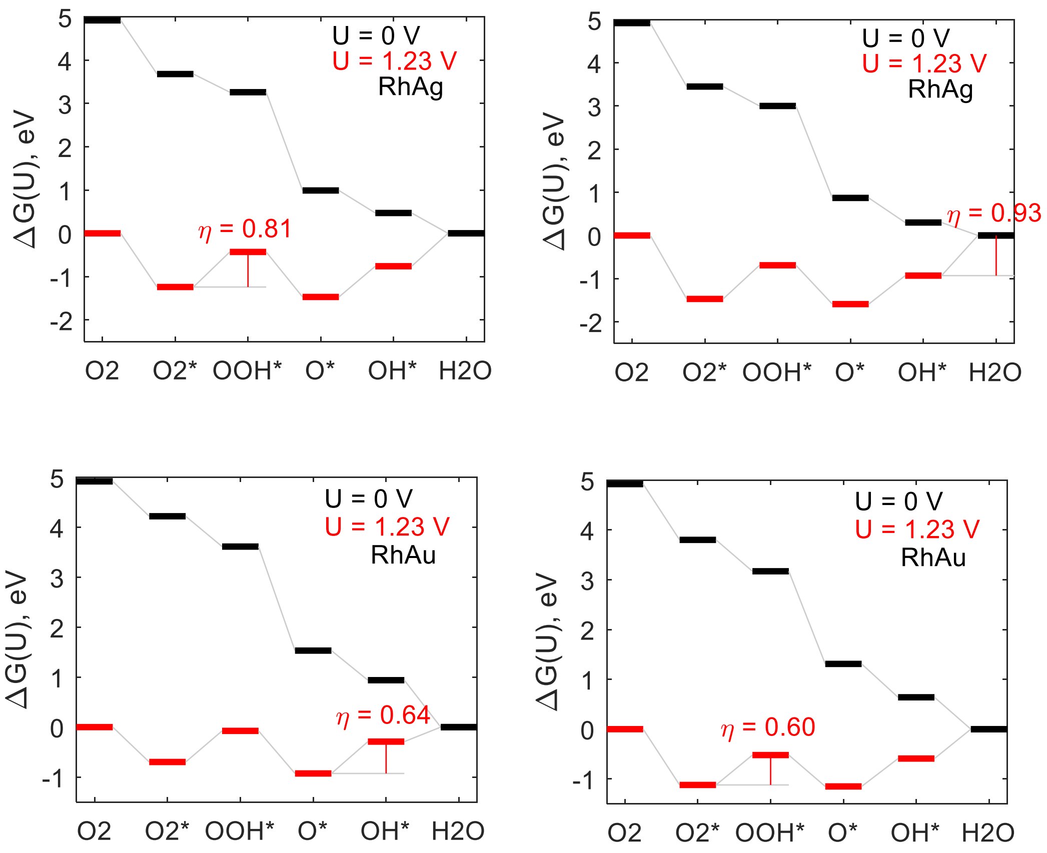

Figure S 1: Potential Gibbs free energy diagrams for the four e^- ORR on SAAs of Co, Ni, Pd, Pt and Rh doped into Ag and Au hosts. Plots are given for each SAA for calculations performed using the computational setup described in the main manuscript (left) and using a D3 dispersion correction (right). Reasonably consistent values are obtained for the calculations with versus without dispersion. Crucially, the best performing SAAs continue to exhibit the lowest overpotentials; the inclusion of the D3 correction yields an unchanged overpotential for PdAu, while for PtAu the overpotential is reduced by 0.09 eV (i.e. this SAA has better predicted performance).

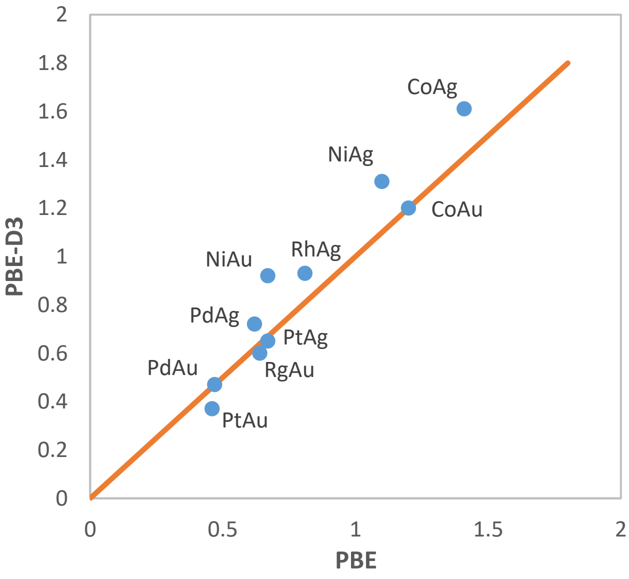

Figure S 2: Parity plot of the overpotentials calculated by PBE (x-axis) versus PBE-D3 (y-axis).
